# Carbimazole-Induced Severe Acquired Aplastic Anemia in a Patient with Graves’ Disease: A Case Report

**DOI:** 10.3390/reports9030287

**Published:** 2026-08-26

**Authors:** Rahaf A. Alghamdi, Hind A. Alshankiti, Adel F. Al-Marzouki, Yara M. Daous

**Affiliations:** 1 Department of Medicine, King Abdulaziz University Hospital, Jeddah 21589, Saudi Arabia; 2 Department of Hematology, King Abdulaziz University Hospital, Jeddah 21589, Saudi Arabia; 3 Hematology Research Unit, King Fahd Medical Research Centre, King Abdulaziz University, Jeddah 21589, Saudi Arabia; 4 Department of Hematology, Faculty of Medicine, King Abdulaziz University, Jeddah 21589, Saudi Arabia; 5 Department of Pathology, Faculty of Medicine, King Abdulaziz University, Jeddah 21589, Saudi Arabia; 6 Department of Pathology, King Abdulaziz University Hospital, Jeddah 21589, Saudi Arabia

**Keywords:** carbimazole, aplastic anemia, Graves’ disease, pancytopenia, bone marrow failure

## Abstract

**Background and Clinical Significance:** Carbimazole is a widely used medication to treat Graves’ disease, although it can rarely cause acquired aplastic anemia, a complication that happens in less than 0.01% of people. **Case Presentation:** We report a 36-year-old woman treated with supratherapeutic dose carbimazole who, after approximately six months of treatment, developed high grade fever, severe menorrhagia, spontaneous epistaxis, and pancytopenia. Her bone marrow biopsy showed severe bone marrow failure with only 5% cellularity and trilineage hypoplasia. Other potential causes were ruled out. Immediate discontinuation of carbimazole was done, and supportive care, including blood transfusions, broad-spectrum antibiotics, G-CSF, and eltrombopag, was started. The patient deteriorated during her hospital stay, developed neutropenic sepsis and acute respiratory failure from diffuse alveolar hemorrhage, which required intubation and pulse steroid therapy. She was stabilized and discharged, with a referral to a tertiary medical center for starting antithymocyte globulin (ATG) immunosuppressive therapy, which she subsequently completed; two months after discharge she was transfusion independent with near-normalization of her blood counts. **Conclusions:** This case serves as a stark reminder of how lethal thionamide-induced bone marrow failure can be, highlighting the vital need for immediate drug cessation, swift intensive care, and thorough patient education on early warning signs.

## 1. Introduction and Clinical Significance

Carbimazole is a widely prescribed antithyroid medication for the treatment of hyperthyroidism, particularly in patients with Graves’ disease, owing to its effectiveness in reducing thyroid hormone synthesis [1]. Although generally well tolerated, carbimazole may rarely cause serious adverse effects, particularly hematologic complications [2,3]. Other medications classically associated with drug induced aplastic anemia include chloramphenicol, sulfonamides, NSAIDs, gold salts, carbamazepine, phenytoin, and other thionamides such as propylthiouracil and methimazole [4,5]. Agranulocytosis is the most recognized severe hematologic toxicity, typically occurring within the first few months of treatment, with an estimated incidence of 0.1–0.5% [2,6]. In contrast, carbimazole-induced aplastic anemia is an exceptionally rare but potentially life-threatening complication.

Aplastic anemia is a bone marrow failure syndrome defined by peripheral blood pancytopenia occurring in conjunction with a hypocellular bone marrow, in the absence of an abnormal infiltrate and with no increase in reticulin fibrosis; severity is graded using standard hematologic criteria based on the degree of neutropenia, thrombocytopenia, and reticulocytopenia [4]. Drug-induced aplastic anemia is characterized by this same picture of pancytopenia and markedly hypocellular bone marrow in the absence of malignant infiltration or marrow fibrosis, arising as an idiosyncratic reaction to an implicated medication [4,5]. Antithyroid drug induced aplastic anemia is exceedingly uncommon, with fewer than 50–60 cases reported worldwide and an estimated incidence of less than 0.01% [2,5,7]. The underlying pathogenesis remains incompletely understood. Current evidence suggests that the condition is primarily immune mediated, involving activated cytotoxic T lymphocytes that target hematopoietic stem and progenitor cells, resulting in profound bone marrow failure [4,8]. Alternatively, direct toxic effects of carbimazole on bone marrow precursor cells have also been proposed [6,9].

Patients typically present with manifestations of pancytopenia, including fatigue secondary to anemia, fever or recurrent infections due to neutropenia, and mucocutaneous bleeding resulting from thrombocytopenia [3,5,7]. Unlike agranulocytosis, which selectively affects neutrophils, aplastic anemia involves failure of all hematopoietic cell lineages and therefore requires prompt recognition, immediate discontinuation of the offending medication, comprehensive hematologic evaluation, and bone marrow examination [1,6]. Delayed diagnosis may lead to life-threatening infectious or hemorrhagic complications. In our patient, this distinction was clinically decisive: her presentation with trilineage cytopenia, rather than isolated neutropenia, was what distinguished her illness from the far more common carbimazole-induced agranulocytosis, and it was this finding that prompted urgent bone marrow biopsy and transfusion support across all three cell lines, rather than antimicrobial therapy alone.

Here, we report a rare case of severe acquired aplastic anemia associated with carbimazole therapy in a patient with Graves’ disease. This case highlights the diagnostic challenges, clinical presentation, hematologic findings, management strategies, and favorable outcome following early recognition and appropriate treatment. It also underscores the importance of maintaining a high index of suspicion for this rare complication in patients receiving antithyroid medications who develop unexplained pancytopenia.

## 2. Case Presentation

### 2.1. Patient Information and Clinical Presentation

The patient was a 36-year-old woman from Pakistan with a known history of Graves’ disease. Six months prior, the patient had begun to experience symptoms of hyperthyroidism, including weight loss, tremors, palpitations, profuse sweating, diffuse goiter, and exophthalmos. She was diagnosed with Graves’ disease at an outside facility and started on carbimazole at a dose of 30 mg three times daily (90 mg/day) as first-line therapy substantially above the standard recommended starting range. The rationale for this initial dose was not documented in the records provided by the referring facility. She remained on 90 mg/day for approximately four months, after which the outside treating team began a taper, first to 10 mg twice daily (20 mg/day) and subsequently to 10 mg once daily (10 mg/day). Two weeks prior to presentation to our care, the patient experienced severe menorrhagia, high grade fever of 39 °C, and spontaneous epistaxis. She presented to an outside hospital, where laboratory testing indicated severe thrombocytopenia with a platelet count of 9 × 10^9^/L (reference range: 150–400 × 10^9^/L), a suppressed TSH (<0.01 mIU/L), elevated free T4 (59.9 pmol/L), and elevated T3 (8.9 nmol/L). She received a supportive transfusion, along with propranolol 300 mg three times daily and hydrocortisone 100 mg intravenously three times daily, without further investigative work-up for the underlying cause of her thrombocytopenia. A thyroid ultrasound performed at that time showed a mildly enlarged thyroid gland with a solitary isthmic nodule (ACR TI-RADS category 3) and diffusely increased vascularity. She was managed as presumed immune thrombocytopenia and discharged on prednisolone 60 mg daily with a taper planned after two weeks; carbimazole was continued at 10 mg daily, on which she remained clinically and biochemically thyrotoxic. Three days prior to presentation to our hospital, she developed a large right forearm ecchymosis, which prompted her to seek further care at our facility.

Upon arrival at our hospital, the patient was hemodynamically stable and afebrile. Her baseline height was 157 cm and weight 82 kg, corresponding to a body mass index of 33.3 kg/m^2^. A diffuse, soft goiter was palpable and visible with the neck in the normal position (WHO grade 2), measuring approximately 4 cm in its greatest dimension, without bruit or retrosternal extension. Marked Graves’ ophthalmopathy was evident clinically; formal exophthalmometry and a Clinical Activity Score were not performed at the time of assessment. Neurological examination was unremarkable. An ecchymosis of approximately 20 × 15 cm was observed on the patient’s right upper arm. There was no mucosal bleeding, lymphadenopathy, hepatosplenomegaly, or an apparent source of infection.

### 2.2. Laboratory Investigations

Laboratory testing revealed pancytopenia involving all three hematopoietic cell lines; of note, the total leukocyte count remained within the normal range despite profound neutropenia. Hemoglobin: 7.5 g/dL (reference range: 12–16 g/dL), mean corpuscular volume (MCV): 78 fL (reference range: 80–100 fL), indicating a mildly microcytic anemia; mean corpuscular hemoglobin: 28 pg, mean corpuscular hemoglobin concentration: 35.9 g/dL, red cell distribution width: 14.2%, with red cells appearing microcytic and normochromic on smear review. Iron studies showed a serum ferritin of 230 µg/L with a transferrin saturation of 7%, a pattern indicating iron-restricted erythropoiesis in the setting of systemic inflammation rather than absolute iron deficiency, ferritin being an acute-phase reactant that may be spuriously preserved in this context, white blood cell count: 4.6 × 10^9^/L (reference range: 4–11 × 10^9^/L), absolute neutrophil count: 0.69 × 10^9^/L, platelet count: 8 × 10^9^/L (reference range: 150–400 × 10^9^/L).

Peripheral smear results showed severe thrombocytopenia and neutropenia with toxic granulation. In addition, 2+ schistocytes and poikilocytosis were observed. Apart from the schistocytes, the smear showed no features of active hemolysis or thrombotic microangiopathy, and this was confirmed biochemically. The lactate dehydrogenase level was 119 U/L (reference range: 140–280 U/L), the total bilirubin level was 5 µmol/L (reference range: 5–21 µmol/L), and the haptoglobin level was 1.64 g/L (reference range: 0.3–2.0 g/L). The reticulocyte count was reduced to 0.0214 M/µL (reference range: 0.025–0.10 M/µL), suggesting a poor marrow response rather than peripheral destruction. In the absence of laboratory evidence of hemolysis, the direct Coombs test was positive; however, it was considered to be clinically insignificant.

Autoimmune work-up included low-titer positive ANA (1:80), while antiphospholipid antibodies, including anti-cardiolipin IgG, anti-cardiolipin IgM, and beta-2 glycoprotein I antibodies, were all negative. ANCA testing, including p-ANCA, C-ANCA, MPO, and PR3 antibodies, was also negative.

Vitamin B12 and folate levels were also obtained, and they showed normal results: 425 pg/mL (reference range: 155–950 pg/mL), 12 ng/mL (reference range: 2.7–17 ng/mL).

Thyroid function tests confirmed persistent hyperthyroidism with suppressed thyroid-stimulating hormone (TSH < 0.0083 mIU/L; reference range: 0.4–4.0 mIU/L), a free T4 within the normal range (15.5 pmol/L; reference range: 9–19 pmol/L), and an elevated total T3 (4.2 nmol/L; reference range: 1.2–2.8 nmol/L), a pattern consistent with predominant T3 toxicosis at the time of admission. Elevated thyroid antibody titers, including anti-thyroglobulin antibodies at 4.05 IU/mL (reference range: 0–4.0 IU/mL) and anti-thyroid peroxidase antibodies at 17.31 IU/mL (reference range: 0–5.6 IU/mL), supported the diagnosis of autoimmune Graves’ disease.

### 2.3. Bone Marrow Examination and Diagnostic Work-Up

Owing to the severity of the patient’s low blood cell counts and the lack of a peripheral cause, an urgent investigation for bone marrow failure was required. A bone marrow aspirate and trephine biopsy were therefore performed on day 7 of admission.

**Figure 1:**
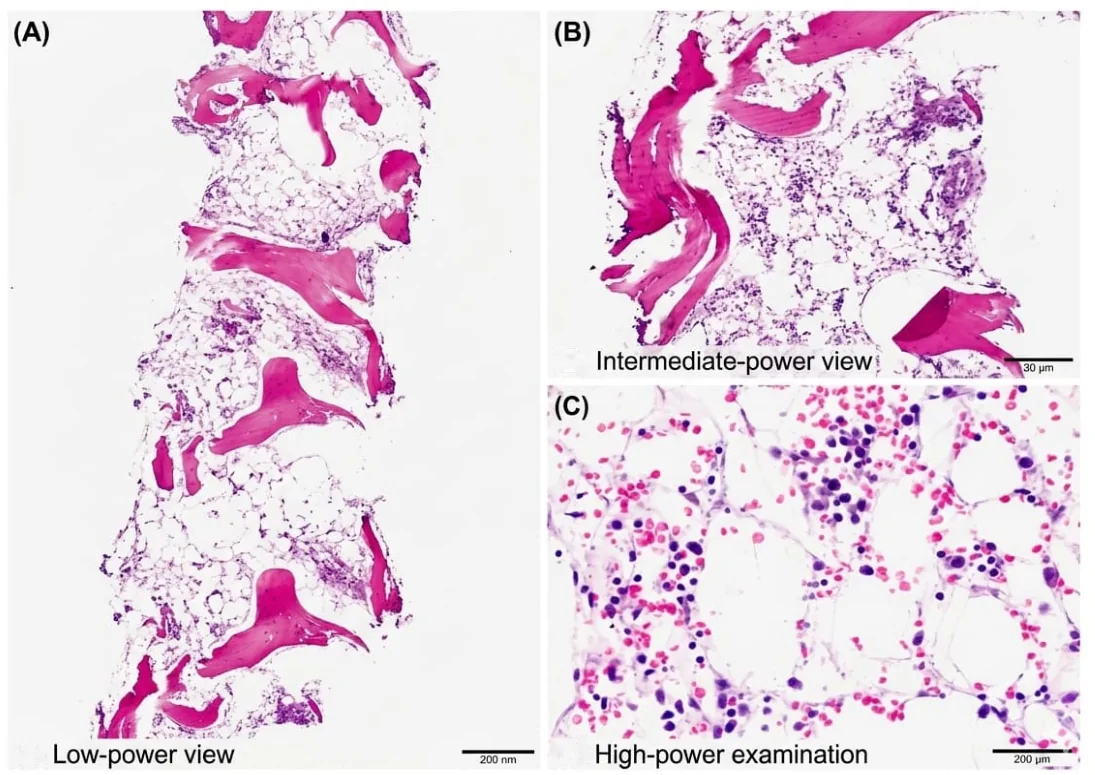
Bone marrow trephine biopsy sections demonstrate a markedly hypocellular marrow characterized by extensive fatty replacement and profound suppression of hematopoiesis. (**A**,**B**) Panel (**A**), low-power view (original magnification × 2), and panel (**B**), intermediate-power view (original magnification × 4), reveal markedly reduced overall marrow cellularity with only small residual foci of hematopoietic tissue. (**C**) High-power view (original magnification × 20) confirms severe trilineage hypoplasia with sparse residual hematopoietic cells and no morphologic evidence of leukemia, metastatic infiltration, granulomatous inflammation, or significant dysplasia. The findings support the diagnosis of aplastic anemia/aplastic crisis.

Bone marrow biopsy revealed profoundly hypocellular marrow with approximately 5% cellularity. There was severe trilineage hypoplasia and a near-complete absence of megakaryocytes. Only a few erythroid and myeloid precursor cells were observed. No dysplasia, fibrosis, infiltrative disease, or blast proliferation was noted. The blast percentage was less than 5%. Flow cytometry revealed no evidence of hematologic malignancy. Cytogenetic analysis revealed a normal female karyotype (46,XX). Based on the severe thrombocytopenia, neutropenia, and reticulocytopenia, the patient fulfilled the known accepted diagnostic criteria for severe acquired aplastic anemia. Testing for paroxysmal nocturnal hemoglobinuria (PNH) was negative. There was a strong temporal association between carbimazole exposure and the development of severe aplastic anemia, as well as exclusion of alternative etiologies, which further supports the diagnosis of carbimazole-induced aplastic anemia. These bone marrow findings are illustrated in Figure 1.

Extensive infectious evaluation was also unremarkable in this case. Hepatitis B core antibody, hepatitis C antibody, human immunodeficiency virus (HIV) types 1 and 2, and hepatitis A immunoglobulin M were negative. Epstein–Barr virus serology showed negative immunoglobulin M with positive immunoglobulin G. Parvovirus B19 serology showed positive immunoglobulin G and negative immunoglobulin M, suggesting prior exposure without evidence of active viral marrow suppression.

All radiological examinations were unremarkable. A brain computed tomography (CT) scan revealed no acute intracranial pathology. Abdominal ultrasound showed a liver size of 11 cm and a spleen size of 12 cm without hepatosplenomegaly. Thyroid ultrasonography revealed the typical appearance of Graves’ disease.

Differential diagnoses considered included viral marrow suppression, hypocellular myelodysplastic syndrome, thrombotic microangiopathy, autoimmune marrow failure, and drug-induced aplastic anemia. However, the absence of dysplastic changes, normal cytogenetics, negative infectious evaluations, and lack of hemolysis or renal impairment make these alternative diagnoses less likely. Viral marrow suppression was excluded by negative parvovirus B19 IgM, negative EBV IgM, and negative HIV and hepatitis serologies, with positive parvovirus and EBV IgG indicating past rather than active infection. Paroxysmal nocturnal hemoglobinuria testing was negative. Thrombotic thrombocytopenic purpura was considered given the scattered schistocytes on peripheral smear, but was felt unlikely based on an intermediate-risk PLASMIC score of 5 together with normal LDH, normal haptoglobin, and the absence of renal impairment or overt hemolysis; ADAMTS13 activity was not available to further refine this assessment. Hypocellular myelodysplastic syndrome was excluded by normal cytogenetics (46,XX) and the absence of dysplastic features on bone marrow examination. Autoimmune marrow failure was considered given a low-titer positive ANA (1:80); however, antiphospholipid antibodies and ANCA panel were negative, and the direct Coombs test, though positive, was not accompanied by biochemical evidence of hemolysis and was considered clinically insignificant. Although Graves’ disease can occasionally be linked to mild cytopenia, the degree of marrow hypocellularity observed in this patient substantially surpassed what would typically be expected as a result of thyrotoxicosis alone.

### 2.4. Treatment and Clinical Outcome

Carbimazole was immediately stopped, and future exposure to carbimazole or alternate thionamide agents was contraindicated due to the risk of cross-reactivity. The patient’s only other recent medication was prednisolone 60 mg daily, initiated at an outside facility approximately two weeks before presentation for presumed immune thrombocytopenia; this was tapered from admission rather than stopped abruptly, while carbimazole was discontinued outright. Glucocorticoids are not recognized as myelosuppressive agents, and no other medications with known marrow-toxic potential were identified in her history. Hyperthyroidism was managed conservatively using propranolol; definitive surgical treatment will be arranged following hematologic stability (Table 1).

The patient required intensive management and received multiple platelet and packed red blood cell transfusions for severe thrombocytopenia and symptomatic anemia. All cellular blood products were leukoreduced and irradiated in accordance with hospital transfusion policy. The patient’s platelet count did recover after the platelet transfusions, but they were never able to reach optimal levels. She was later neutropenic from admission, and empiric ceftazidime was started, this was discontinued after several days following a negative infectious workup, including the absence of other spikes of fever. Three weeks later, during a period of worsening pancytopenia, she developed genuine neutropenic fever with chills and temperatures up to 38 °C, and was started on intravenous meropenem and caspofungin. Tranexamic acid for bleeding control was started early in her admission. Granulocyte colony-stimulating factor (G-CSF), 300 mcg daily, was commenced on day 7 of admission and given for 7 days to support neutrophil recovery. Eltrombopag 50 mg daily was later commenced on day 32, three days prior to her transfer to the tertiary center, and was continued as a bridge to definitive therapy until ATG-based immunosuppression was started.

Initially, the patient appeared to have a partial response based on her blood test results. However, even after receiving additional supportive care, she continued to have repeated episodes of severe thrombocytopenia and ongoing bleeding, which suggested persistent aplastic disease rather than transient drug-induced suppression.

**Figure 2:**
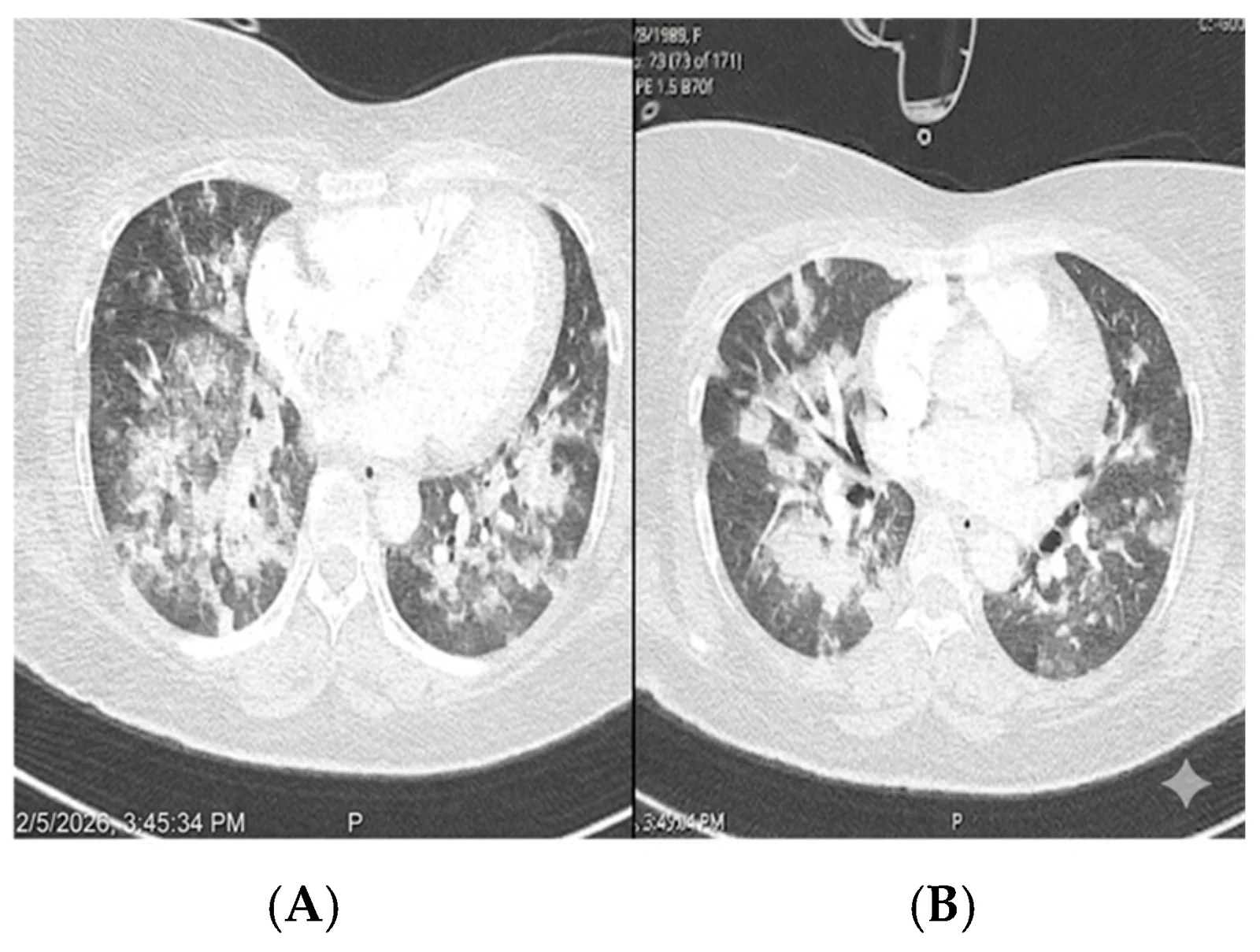
High-resolution chest CT scan (HRCT) slices. (**A**,**B**) Axial views demonstrate bilateral, multifocal peribronchovascular consolidations superimposed on widespread ground-glass opacities (GGO). Given the clinical history and acute development, these findings are highly suggestive of diffuse alveolar hemorrhage.

On day 28 of admission the patient suddenly developed difficulty breathing associated with hemoptysis. A rapid response team was called, and the patient was intubated to protect her airway and required mechanical ventilation for four days, with the respiratory episode lasting five days in total. A high-resolution chest CT scan showed signs of diffuse alveolar hemorrhage or an inflammatory vasculitic process. Consequently, her antibiotic regimen was escalated to include meropenem, vancomycin, and fluconazole. The patient also received high-dose methylprednisolone. At that time, she was already taking prednisone (1 mg/kg) along with thrombopoietin receptor agonist therapy. The corresponding HRCT findings are shown in Figure 2.

Although her blood counts slowly improved, she required blood transfusions throughout her hospital stay. She was discharged in a stable condition after a 35-day admission and continued to be followed up by the hematology team. During outpatient follow-up, she was referred to a tertiary center for further management with antithymocyte globulin (ATG)-based immunosuppressive therapy. Allogeneic bone marrow transplantation was not performed; it was identified as a contingency option to be pursued only in the event of inadequate long term hematologic recovery with ATG-based therapy. ATG-based immunosuppressive therapy was commenced three weeks after discharge and the full course was completed. At the most recent follow-up, two months after discharge, her hemoglobin was 12.1 g/dL, absolute neutrophil count 3.2 × 10^9^/L, and platelet count 79 × 10^9^/L, and she had been transfusion independent for three weeks. Definitive management of her Graves’ disease with radioiodine is planned once hematologic recovery is sustained. She reported a marked improvement in her overall condition and had returned to her usual daily activities.

### 2.5. Patient Perspective

I was very frightened when all of this began. I had never liked the way my neck looked, and it had been getting bigger; then the bruises started appearing on my body for no reason at all, and they kept coming. I was bleeding heavily and had a high fever, and I did not understand what was happening to me. The medicine I was taking made me feel unwell, and I began to lose trust in the doctors and in the treatment I had been given, because no one had explained to me why I was getting worse instead of better. When the doctors at this hospital examined me, did the bone marrow test and told me clearly that the medication had caused my blood counts to fall, I finally understood my illness. Having a name for what was wrong with me, and knowing that the drug had been stopped, made me feel much calmer and safer. I am grateful for the care I received, I feel more comfortable and hopeful now, and I am encouraged to continue with the rest of my treatment.

## 3. Discussion

This case describes a rare but serious and life-threatening side effect of carbimazole-induced aplastic anemia. Agranulocytosis is the most common serious hematologic adverse effect of antithyroid drugs, whereas aplastic anemia is considerably rarer and more serious because it affects all the bone marrow lines [5,7,10]. Our patient presented with typical signs of severe aplastic anemia. These included symptomatic anemia, thrombocytopenia associated bleeding, and neutropenia associated fever. Other reported cases of this condition have shown similar symptoms [5,7,10]. The timing of presentation in this case was similar to that in previous reports. Most cases occur within the first few months of starting the drug [5,7]. Importantly, it remains unpredictable who will develop this complication, as it does not seem to follow a clear dose–response relationship. This suggests an idiosyncratic immune mediated mechanism rather than cumulative toxicity alone, although sustained high dose exposure may have acted as a contributory risk factor in our patient [2,9].

Standard guidelines recommend carbimazole doses of 10–40 mg daily for initial control of thyrotoxicosis, with maintenance doses typically reduced to 5–15 mg daily once euthyroidism is achieved [11]. Our patient was initially started on 30 mg three times daily (90 mg/day) at an outside facility, more than double the upper limit of the standard recommended range, and remained on this dose for approximately four months before a taper was begun by the referring team. While supratherapeutic doses may achieve faster biochemical control of severe thyrotoxicosis, there is no clear evidence that doses this far above the standard range confer additional clinical benefit. A dose-dependent relationship for this reaction has not been established in the literature, and the mechanism is generally regarded as idiosyncratic rather than dose-driven; nonetheless, the prolonged exposure to a markedly supratherapeutic dose in our patient cannot be excluded as a contributing factor to the severity of her marrow toxicity [2,9]. more conservative initial dosing, with closer titration to the lowest effective dose, might have reduced, although not necessarily eliminated, the risk.

Bone marrow trephine biopsy is a key diagnostic component in the evaluation of aplastic anemia, as it confirms marrow hypocellularity and helps exclude infiltrative, dysplastic, or fibrotic marrow disorders that may mimic the condition. In our patient, trephine biopsy confirmed a severely hypocellular marrow (approximately 5% cellularity) with marked trilineage hypoplasia, rare scattered erythroid and myeloid precursors, and virtually absent megakaryocytes; residual cellularity consisted predominantly of small, mature lymphocytes and plasma cells. Iron and reticulin stains showed mildly increased storage iron without an increase in reticulin fibers, arguing against a fibrotic marrow process. Immunohistochemistry (CD34, MPO, E-cadherin, CD3, CD20, CD138) confirmed no increase in blasts and no aberrant infiltrate, with CD3 and CD20 highlighting a mixed population of T and B lymphocytes without morphologic features suggestive of a clonal lymphoid process findings that establish the marrow morphology of aplastic anemia and exclude hypocellular myelodysplastic syndrome and marrow infiltration by malignancy; taken together with the peripheral blood parameters described in Section 2.2 (severe neutropenia, thrombocytopenia, and reticulocytopenia), these findings fulfilled criteria for severe aplastic anemia. These findings closely mirror those reported in other published cases of thionamide induced aplastic anemia [4,5,10]. Although the exact pathogenesis remains uncertain, evidence from the broader aplastic anemia literature points to immune mediated destruction of hematopoietic stem and progenitor cells by activated cytotoxic T lymphocytes [4,8]; while marrow immunophenotyping in our patient demonstrated a T-lymphocyte population, this finding alone does not establish that this mechanism was operative in her case. This theory is further supported by reports of abnormal T-cell populations and cytokine dysregulation in affected patients more broadly. Direct toxic effects of carbimazole on marrow precursor cells have also been proposed, though this mechanism does not readily account for the marked interpatient variability in presentation and severity [6,9]. A major diagnostic challenge in this case was systematically excluding alternative causes of pancytopenia with a similar morphologic appearance: viral associated marrow suppression, paroxysmal nocturnal hemoglobinuria, thrombotic microangiopathy, and hypocellular myelodysplastic syndrome were each excluded through serologic testing, flow cytometry, PLASMIC scoring, cytogenetic analysis, and bone marrow immunohistochemistry, as detailed in Section 2.3. Graves’ disease itself can rarely cause mild, usually isolated cytopenia, but the degree of trilineage marrow failure observed here far exceeds what would be expected from thyrotoxicosis alone [12].

The treatment of drug induced aplastic anemia begins with the immediate discontinuation of the offending medication. Patients also require aggressive supportive care, such as blood transfusions and antibiotics [1,6]. Re-exposure to carbimazole or alternative thionamides should be avoided because of the risk of recurrence and cross-reactivity [6,11,13]. Given the severity of her disease and its complications, our patient received repeated blood transfusions and broad-spectrum antimicrobial therapy, along with G-CSF (300 mcg daily for 7 days) as adjunctive support for her neutropenia and, later, eltrombopag (50 mg daily) as a bridging measure while awaiting definitive immunosuppressive therapy. Recent studies have shown that eltrombopag, used alongside immunosuppressive therapy rather than as a stand-alone treatment, may help bone marrow recovery in difficult cases of severe aplastic anemia [14]. ATG-based immunosuppressive therapy, which our patient subsequently received, remains the standard definitive approach for severe aplastic anemia in patients who are not transplant candidates, and its efficacy further supports the role of immune-mediated marrow injury in this disease [8,15]. Compared with previously reported cases of antithyroid drug-induced aplastic anemia, which have generally been managed with drug withdrawal and supportive transfusion alone, our patient’s course was notably more severe, marked by neutropenic sepsis and diffuse alveolar hemorrhage requiring intensive care and mechanical ventilation. Reported outcomes in the literature range from full hematologic recovery after drug cessation to fatal outcomes in cases complicated by severe infection or hemorrhage [5,7,10]. Our patient’s survival to hospital discharge, despite this severe complication, together with substantial hematologic recovery by two months after discharge following combined supportive therapy and ATG-based immunosuppression, is broadly consistent with the more favorable end of previously reported outcomes, though her prolonged and complicated course underscores the potential severity of this condition even with early drug discontinuation and aggressive supportive care.

Even with good supportive care, severe aplastic anemia remains a serious condition. It can cause life threatening complications, such as neutropenic sepsis and diffuse alveolar hemorrhage. Our patient had both complications. Similar complications have been reported in other cases of antithyroid drug-induced aplastic anemia [3,6]. This case also highlights the importance of educating patients taking antithyroid medications about warning signs. These include fever, sore throat, bleeding, and unexplained bruising. Medical evaluation and immediate discontinuation of the offending medication remain essential if these symptoms appear; because reactions of this kind can develop abruptly between visits, scheduled routine blood-count monitoring, while still valuable, cannot by itself be relied upon to catch every episode, and patient education on warning symptoms should accompany rather than replace it [3,6]. Important knowledge gaps remain in this area. No validated risk-prediction model currently exists to identify which patients on thionamide therapy are at risk of aplastic anemia rather than the more common agranulocytosis, and potential genetic associations (for example, HLA subtypes linked to other forms of drug-induced marrow toxicity) have not been systematically studied for this specific complication. Prognostic factors that determine disease severity and recovery such as degree of marrow hypocellularity, timing of drug cessation, and response to immunosuppression are not well characterized due to the small number of reported cases. There is also no standardized management protocol specific to thionamide-induced aplastic anemia, with current practice extrapolated largely from general aplastic anemia guidelines [15]. Given the rarity of this complication, multicenter case registries and pooled analyses of published cases would be valuable in establishing clearer risk factors, prognostic markers, and standardized treatment pathways.

## 4. Conclusions

Carbimazole-induced aplastic anemia is a rare but potentially fatal complication of antithyroid therapy. Clinicians should maintain a high index of suspicion in patients receiving thionamides who develop pancytopenia, bleeding manifestations, or infectious symptoms during treatment. Early recognition, discontinuation of the offending medication, urgent bone marrow evaluation, and aggressive supportive management are critical for improving outcomes. This case adds to the limited literature on carbimazole-induced aplastic anemia and emphasizes the importance of awareness of this serious hematologic complication.

## Figures and Tables

**Table 1:** Chronological sequence of clinical events and treatment during the hospital admission. Day 0 denotes the day of admission to our hospital.

Day	Clinical Event	Intervention and Duration
−180	Diagnosis of Graves’ disease at an outside facility	Carbimazole 30 mg three times daily (90 mg/day) commenced
−180 to −60	Carbimazole continued at 90 mg/day	No dose reduction during this period
−60	Outpatient review at the referring facility	Carbimazole reduced to 10 mg twice daily (20 mg/day)
−30	Further outpatient review	Carbimazole reduced to 10 mg once daily (10 mg/day)
−14	Onset of menorrhagia, fever 39 °C and epistaxis; platelets 9 × 10^9^/L	Platelet transfusion, propranolol and intravenous hydrocortisone given; carbimazole continued at 10 mg/day; discharged on prednisolone 60 mg daily
−3	Large right forearm ecchymosis	Presented to our facility
0	Admission; pancytopenia with ANC 0.69 × 10^9^/L and platelets 8 × 10^9^/L	Carbimazole stopped; prednisolone tapered from admission; propranolol continued; packed red cell and platelet transfusions and tranexamic acid commenced
12	Bone marrow aspirate and trephine biopsy performed	Diagnosis of severe acquired aplastic anemia confirmed
0 and 21	Neutropenic fever	Ceftazidime (Day 0–4); meropenem and caspofungin (Day 21)
7 and 32	Persistent severe thrombocytopenia and bleeding	G-CSF 300 mcg daily commenced on day 7, given for 7 days; eltrombopag 50 mg daily commenced on day 32, three days before transfer to the tertiary center, and continued until ATG-based therapy was started
28	Acute respiratory failure with hemoptysis; HRCT consistent with diffuse alveolar hemorrhage	Respiratory episode lasted 5 days with 4 days of mechanical ventilation; antimicrobials escalated to meropenem, vancomycin and fluconazole; pulse methylprednisolone administered
31	Extubation and clinical stabilization	Weaned from ventilation; corticosteroids subsequently tapered
35	Discharge	Discharged after a 35-day admission; outpatient hematology follow-up and referral for ATG-based immunosuppressive therapy

## Data Availability

The original data presented in the study are included in the article, further inquiries can be directed to the corresponding author.

## Abbreviations

The following abbreviations are used in this manuscript:

ANA	Antinuclear Antibody
ANC	Absolute Neutrophil Count
ANCA	Antineutrophil Cytoplasmic Antibody
ATG	Antithymocyte Globulin
C-ANCA	Cytoplasmic Antineutrophil Cytoplasmic Antibody
CT	Computed Tomography
G-CSF	Granulocyte Colony-Stimulating Factor
GGO	Ground-Glass Opacities
HIV	Human Immunodeficiency Virus
HRCT	High-Resolution Computed Tomography
IgG	Immunoglobulin G
IgM	Immunoglobulin M
MPO	Myeloperoxidase
PNH	Paroxysmal Nocturnal Hemoglobinuria
PR3	Proteinase 3
p-ANCA	Perinuclear Antineutrophil Cytoplasmic Antibody
T3	Triiodothyronine
T4	Thyroxine
TSH	Thyroid-Stimulating Hormone
